# An efficient co-culture of *Halomonas mongoliensis* and *Dunaliella salina* for phenol degradation under high salt conditions

**DOI:** 10.3389/fmicb.2024.1505542

**Published:** 2024-12-11

**Authors:** Changjian Wang, Haiqiao Guo, Peng Yu, Bo Huang, Zhikun Xin, Xufan Zheng, Jinli Zhang, Tao Tang

**Affiliations:** ^1^CHN Energy BaoRiXiLe Energy Co., Ltd., Hulunbeier, China; ^2^School of Civil and Resources Engineering, Graduate School of University of Science and Technology Beijing, Beijing, China; ^3^CHN Energy New Energy Technology Research Institute Co., Ltd., Beijing, China; ^4^CAS Key Lab of Low-Carbon Conversion Science and Engineering, Shanghai Advanced Research Institute, Chinese Academy of Sciences, Shanghai, China; ^5^State Key Laboratory of Low Carbon Catalysis and Carbon Dioxide Utilization, Shanghai Advanced Research Institute, Chinese Academy of Sciences, Shanghai, China

**Keywords:** phenol removal, *Dunaliella salina*, *Halomonas mongoliensis*, co-culture system, adaptive laboratory evolution

## Abstract

Phenol is one of the major organic pollutants in high salt industrial wastewater. The biological treatment method is considered to be a cost-effective and eco-friendly method, in which the co-culture of microalgae and bacteria shows a number of advantages. In the previous study, a co-culture system featuring *Dunaliella salina* (*D. salina*) and *Halomonas mongoliensis* (*H. mongoliensis*) was established and could degrade 400 mg L^−1^ phenol at 3% NaCl concentration. In order to enhance the performance of this system, *D. salina* strain was subjected to adaptive laboratory evolution (ALE) by gradually increasing the phenol concentration from 200 mg L^−1^ to 500 mg L^−1^ at 3% NaCl concentration. At a phenol concentration of 500 mg L^−1^, the phenol removal rate of the resulting *D. salina* was 78.4% within 7 days, while that of the original strain was only 49.2%. The SOD, POD, and MDA contents of the resulting strain were lower than those of the original strain, indicating that the high concentration of phenol was less harmful to the resulting strain. A co-culture system was established with the resulting *D. salina* and *H. mongoliensis*, which could complete degrade 500 mg L^−1^ of phenol within 8 days, outperforming the original *D. salina* co-culture system. This study proved that ALE could improve the phenol tolerance and phenol degradation capability of *D. salina*, and then effectively improve the phenol degradation capability of *D. salina* and *H. mongoliensis* co-culture system.

## 1 Introduction

Industrial processes, such as oil processing and steel production, produce high concentrations of phenol-containing wastewater. It is estimated that 5% of industrial wastewater is saline or hypersaline, which are defined as waters containing dissolved salts (mainly NaCl) in excess of 1 and 3.5% (w/v), respectively (Li et al., 2019). Traditional wastewater treatment techniques, typically physical and chemical treatment methods, have been developed for the removal of phenol (Mohammadi et al., 2015). However, these methods are often costly and can lead to secondary contamination (Mohsenpour et al., 2021). Therefore, economical and environmental treatment technologies should be developed.

Compared with physical and chemical treatment methods, biological treatment (Bhattacharya et al., 2018; Ceylan et al., 2011) has been demonstrated as an environmentally friendly phenol removal method. These microorganisms often include fungi, microalgae and bacteria. At the same time, the emerging concept of microalgae and bacteria consortium has attracted attention. Molecular oxygen produced by microalgae can be used by aerobic bacteria as electron acceptors to degrade certain organic matter, while carbon dioxide released during mineralization completes the photosynthetic cycle (Borde et al., 2003). Therefore, the microalgae and bacteria consortium show a number of advantages over individual microalgae or bacteria, including efficient pollutant removal, high value-added microalgae, cost-effective aeration and reduced greenhouse gas emissions (Su et al., 2012).

The enhancement of phenol degradation by co-culture of microalgae and bacteria has been demonstrated by many reports. One biofilm was developed by Maza-Márquez et al. (2017), which is composed of microalgae, cyanobacteria and bacteria. The biofilm was used to remove phenolic compounds from olive washing water, and the removal rate was higher than 90%. Yi et al. (2020) reported that the growth of *Chlorella* sp. was seriously suppressed and could not degrade 400 mg L^−1^ phenol. However, 1,200 mg L^−1^ phenol could be degraded by the co-culture of *Chlorella* sp. and *C. necator* within 60 h. The co-culture of microalgae and bacteria have shown great potential for phenol wastewater treatment. However, there are only few studies were reported for phenol wastewater with high salinity (Zhang et al., 2022).

In a previous study (Zhang et al., 2022), a co-culture system featuring *D. salina* and *H. mongoliensis* was established. This system successfully degraded 400 mg L^−1^ of phenol within 5 days under 3% NaCl conditions. Generally, bacteria have much higher phenol tolerance and degradation capability than microalgae (Mohammadi et al., 2015; Bhattacharya et al., 2018). Therefore, compared to enhance the phenol degradation capacity of bacteria, it is more significant to improve the phenol tolerance and phenol degradation capability of microalgae under high phenol concentration (Yi et al., 2020). If the phenol tolerance and phenol degradation capability of microalgae are enhanced, the early initiation of photosynthesis can improve synergistic effect between microalgae and bacteria, thus speeding up the degradation rate of phenol and obtaining more microalgae biomass.

Adaptive Laboratory Evolution (ALE) has been demonstrated and often used for microalgal strain selection to improve microalgal growth rate, tolerance, substrate utilization and product yield (LaPanse et al., 2021; Zhang et al., 2021). ALE was also carried out to enhance the tolerance to high salt concentration (Perrineau et al., 2014; Puranik et al., 2012) or the ability to degrade high concentrations of phenol (Wang et al., 2016). Wang et al. (2016) reported the adaptation of a *Chlorella* sp. to high concentrations of phenol (700 mg L^−1^). The resulting strain doubled its maximal biomass concentration, in contrast to the wild type, while removing up to 500 mg L^−1^ of phenol in 7 days. Li et al. (2015) reported one evolved *Chlorella* sp. AE10 strain was obtained by ALE method under 10% CO_2_ (Li et al., 2015). ALE was further conducted on *Chlorella* sp. AE10 under 30 g L^−1^ salt, and the resulting strain *Chlorella* sp. S30 was obtained after 46 cycles of ALE (Li et al., 2018). The resulting strain *Chlorella* sp. S30 survived under 10% CO_2_ and 30 g L^−1^ salt conditions, and the final biomass concentration was 2.7 g L^−1^. It was proved that it was possible to construct an evolved strain with two tolerance abilities for high salinity and high concentration of CO_2_ (Li et al., 2018).

In our previous study (Zhang et al., 2022), a co-culture system featuring the marine microalga *D. salina* and the phenol-degrading bacterium *H. mongoliensis* has been established for phenol degradation at 3% NaCl concentration. In order to build a more efficient *D. salina* and *H. mongoliensis* co-culture system, with a constant NaCl concentration of 3%, ALE was conducted to improve the degradation capacity of *D. salina* to high concentration of phenol. The resulting *D. salina* strain, along with *H. mongoliensis*, was then used to create new co-culture system, and the phenol degradation capacity of the new co-culture system was studied in detail.

## 2 Experiment

### 2.1 Organisms and culture conditions

The microalgal and bacterial strains are *D. salina* and *H. mongoliensis*, respectively. The source, preservation and cultivation methods of microalgae and bacteria are consistent with our previous published study (Zhang et al., 2022). The marine microalga *D. salina* was obtained from Shanghai Guangyu Biological Technology Co., LTD and cultured in petri dishes using BG11 solid medium with 3% NaCl. Subsequently, *D. salina* cells were transferred to 250 mL flasks and further cultivated in 400 mL bubble column photobioreactors under conditions of 1% CO_2_, 120 μmol m^−2^ s^−1^ light intensity, and a temperature of 25°C. The phenol-degrading bacterium *H. mongoliensis* (No. 1.7454) was sourced from the China General Microbiological Culture Collection Center and maintained in petri dishes on 2216E solid culture medium. *H. mongoliensis* was then transferred to 250 mL flasks and incubated on a rotary shaker at 30°C and 150 rpm. During their logarithmic growth phase, the cells of both *D. salina* and *H. mongoliensis* were harvested by centrifugation. The collected cells were re-suspended in simulated phenol wastewater at the desired biomass density and phenol concentration for subsequent experiments. A stock solution of phenol (2,000 mg L^−1^) was prepared by dissolving phenol in sterilized BG11 medium containing 3% NaCl, and the required phenol concentrations were achieved by diluting this stock solution with the same sterilized medium.

### 2.2 ALE

With a constant NaCl concentration of 3%, ALE was conducted on the original *D. salina* strain using a strategy that gradually increased phenol concentrations. The process consisted of four stages: during the first stage (cycles 1–12), the phenol concentration was set at 200 mg L^−1^. In the second stage (cycles 13–24), the phenol concentration rose to 300 mg L^−1^. The third stage (cycles 25–55) involved a further increase to 400 mg L^−1^ phenol, and in the final stage (cycles 56–70), the phenol concentration reached 500 mg L^−1^. Throughout each cycle, the initial cell density was consistently maintained at approximately 0.25 g L^−1^. *D. salina* was cultivated in 250 mL glass flasks containing 100 mL of simulated phenol wastewater, incubated at 30°C on a rotary shaker at 150 rpm. Each ALE cycle lasted 3 days, and at the end, biomass concentration was determined using the dry weight method. Cells were then centrifuged at 6,000 rpm for 5 min, washed twice with sterilized water, and re-suspended in fresh simulated phenol wastewater to initiate the next cycle. All experiments were performed in triplicate, leading to the development of a new strain after 210 days.

### 2.3 Characterization of the resulting strain

To evaluate the phenol degradation capabilities, both the resulting *D. salina* strain and the original strain were cultivated in 250 mL glass flasks with 100 mL of simulated phenol wastewater, incubated at 30°C on a rotary shaker at 150 rpm. The initial cell density was set at 0.2 g L^−1^, and phenol concentrations were tested at 100, 300, and 500 mg L^−1^. Residual phenol levels in the culture medium were assessed using the colorimetric assay based on the 4-amino antipyrine method (Zhou et al., 2017). Growth rates were measured via dry weight (Chandra et al., 2016), while the Fv/Fm values (García-Canedo et al., 2016), indicating the maximum quantum yield of photosystem II, were recorded daily. The dynamic responses of antioxidative enzyme activities in both strains under 500 mg L^−1^ phenol were investigated by collecting 10 mL samples at various time points (initial, 1st, 3rd, 5th, and 7th days). After centrifugation at 6,000 rpm for 5 min, the cell pellets were washed and homogenized with 1 mL of 20 mM phosphate buffer (pH 7.4) and 0.1 g of white quartz sand. The supernatant was obtained after centrifugation at 12,000 rpm for 10 min at 4°C for enzyme activity assays, which were standardized to protein content. Superoxide dismutase (SOD) activity was measured using a SOD assay kit (Sigma, United States) with WST-1 reagent. Catalase (CAT) and peroxidase (POD) activities were evaluated using their respective assay kits (Nanjing Jiancheng Bioengineering Institute, China). Total protein concentration was quantified using a BCA kit (Sangon, China). Malondialdehyde (MDA) extraction followed the same protocol as protein extraction but used potassium phosphate buffer (pH 7.0) for the extraction solution, with MDA content measured using a dedicated assay kit.

### 2.4 Co-culture of *Halomonas mongoliensis* and the resulting *Dunaliella salina* strain

*H. mongoliensis* was co-cultivated with the resulting *D. salina* strain to assess the impacts of phenol degradation and *D. salina* growth across different phenol concentrations. Initial phenol levels were adjusted to 300, 500, and 700 mg L^−1^ using a stock solution. The experiments took place in 500 mL conical flasks (with a working volume of 300 mL), fitted with breathable sealing membranes and shaken at 150 rpm, under 110 μmol m^−2^ s^−1^ light intensity at 25°C. The initial concentrations for *D. salina* and *H. mongoliensis* were 0.2 and 0.1 g L^−1^, respectively, and the pH was adjusted to 7.5 with a 1 mol L^−1^ NaOH solution. Control experiments involved co-cultivating *H. mongoliensis* with the original *D. salina* strain under the same conditions, with all tests conducted in duplicate.

### 2.5 Analysis methods

Ten milliliter samples were taken every day from each flask to assess residual phenol concentration, biomass concentration, and Fv/Fm values. The maximum quantum yield of photosystem II was determined using 2 mL of each sample, stored in the dark for 30 min before measurement with a fluorescence monitoring system (FMS2, Lufthansa Scientific Instruments Co., Ltd., UK). To measure residual phenol, 1 mL of the sample was centrifuged at 6,000 rpm for 10 min, and the supernatant was analyzed using the 4-amino antipyrine method (Zhou et al., 2017). Phenol removal efficiency (RE%) was calculated using Equation 1:


(1) $$RE\left(\%\right)=\left(C_{i}-C_{t}\right)/C_{i}\times 100$$

Where, RE (%) is the removal efficiency of phenol. *C_i_* and *C_t_* are the concentrations of phenol at the initial stage and after the indicated time, respectively.

The biomass concentrations of *D. salina* in monocultures were determined gravimetrically. A 5 mL sample was passed through a pre-dried and pre-weighed cellulose membrane (with a pore size of 0.45 μm). After filtering, the membrane was washed with deionized water, dried for 24 h at 105°C, cooled in a desiccator, and then weighed again. The biomass was obtained by subtracting the dry weight of the blank filter from that of the loaded filter.

In co-culture scenarios, the biomass concentration of *D. salina* was measured indirectly by assessing the concentrations of chlorophyll a and b (Chl a + b). Following the method outlined by Russel et al. (2020), a 0.5 mL sample was centrifuged at 13,400 rpm for 10 min, and the supernatant was discarded. The pellets were then treated with 1.5 mL of methanol to extract chlorophyll a and b, which were quantified as described by Zhang et al. (2022). The concentrations of Chl a + b were calculated using Equations 2–4:


(2) $$\begin{array}{l} Chl\;a=[-8.0962\times \left(OD_{652}-OD_{750}\right)\\ \;+16.5169\times \left(OD_{665}-OD_{750}\right)]\times 3 \end{array}$$

(3) $$\begin{array}{l} Chl\;b=[27.4405\times \left(OD_{652}-OD_{750}\right)\\ \;-12.1688\times \left(OD_{665}-OD_{750}\right)]\times 3 \end{array}$$

(4) Chla+b=Chla+Chlb

Where Chl a, Chl b, and Chl a + b are the concentrations of chlorophyll a (mg L^−1^), chlorophyll b (mg L^−1^) and chlorophyll a and b (mg L^−1^), respectively. OD_652_, OD_665_ and OD_750_ are the optical densities of the extraction solution at wavelengths of 652, 665 and 750 nm, respectively.

## 3 Results and discussion

### 3.1 Adaptive laboratory evolution

**Figure 1:**
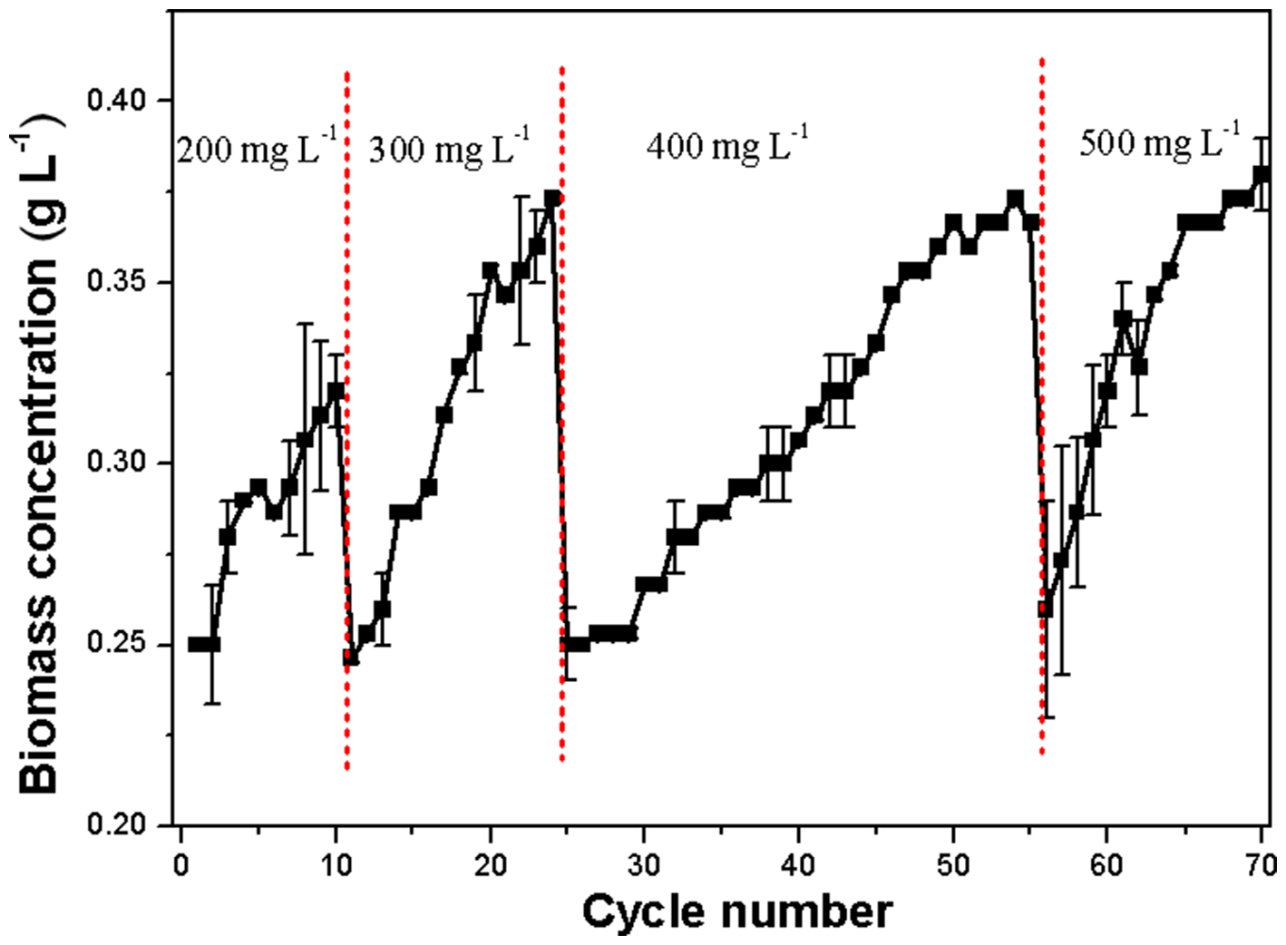
Growth profile of *D. salina* under 200–500 mg L^−1^ phenol during ALE from 1 to 70 cycles.

Previous research indicated that high phenol concentrations (300–500 mg L^−1^) significantly harmed *D. salina* under high salt conditions, impacting its growth and survival (Zhang et al., 2022). This study introduces an ALE approach to enhance phenol tolerance and degradation capacities of *D. salina*. The effectiveness of ALE is influenced by the selected environmental pressures. Excessive stress can hinder evolution, while too little can prolong adaptation. It was observed that *D. salina* could fully degrade 100 mg L^−1^ phenol within 5 days, but removal efficiency dropped to 75.5, 50.6, 30.3, and 27.3% as phenol concentrations rose to 200, 300, 400, and 500 mg L^−1^, respectively (Zhang et al., 2022). Thus, 200 mg L^−1^ was chosen as the initial concentration, with a stepwise increase implemented over four stages. As shown in Figure 1, in the first stage (cycles 1–10), the strain tolerated 200 mg L^−1^ phenol, reaching a biomass concentration of 0.32 g L^−1^. In the second stage (cycles 11–24), the phenol concentration increased to 300 mg L^−1^, with biomass reaching 0.37 g L^−1^ by cycle 24. During the third stage (cycles 25–55) at 400 mg L^−1^ phenol, the adaptive evolution rate slowed, requiring about 2.2 times longer to achieve similar biomass levels as in the second phase. Finally, in the fourth stage, at 500 mg L^−1^ phenol, the strain adapted quickly, reaching 0.38 g L^−1^ by cycle 70. The resulting *D. salina* strain with enhanced phenol tolerance was successfully developed after 70 cycles (210 days). Initially low biomass levels increased over time, confirming the effectiveness of the ALE process in selecting phenol-tolerant phenotypes, similar to findings with *C. reinhardtii* (Yu et al., 2013) and *C. vulgaris* (Fu et al., 2013).

### 3.2 Characterization of the resulting strain

**Figure 2:**
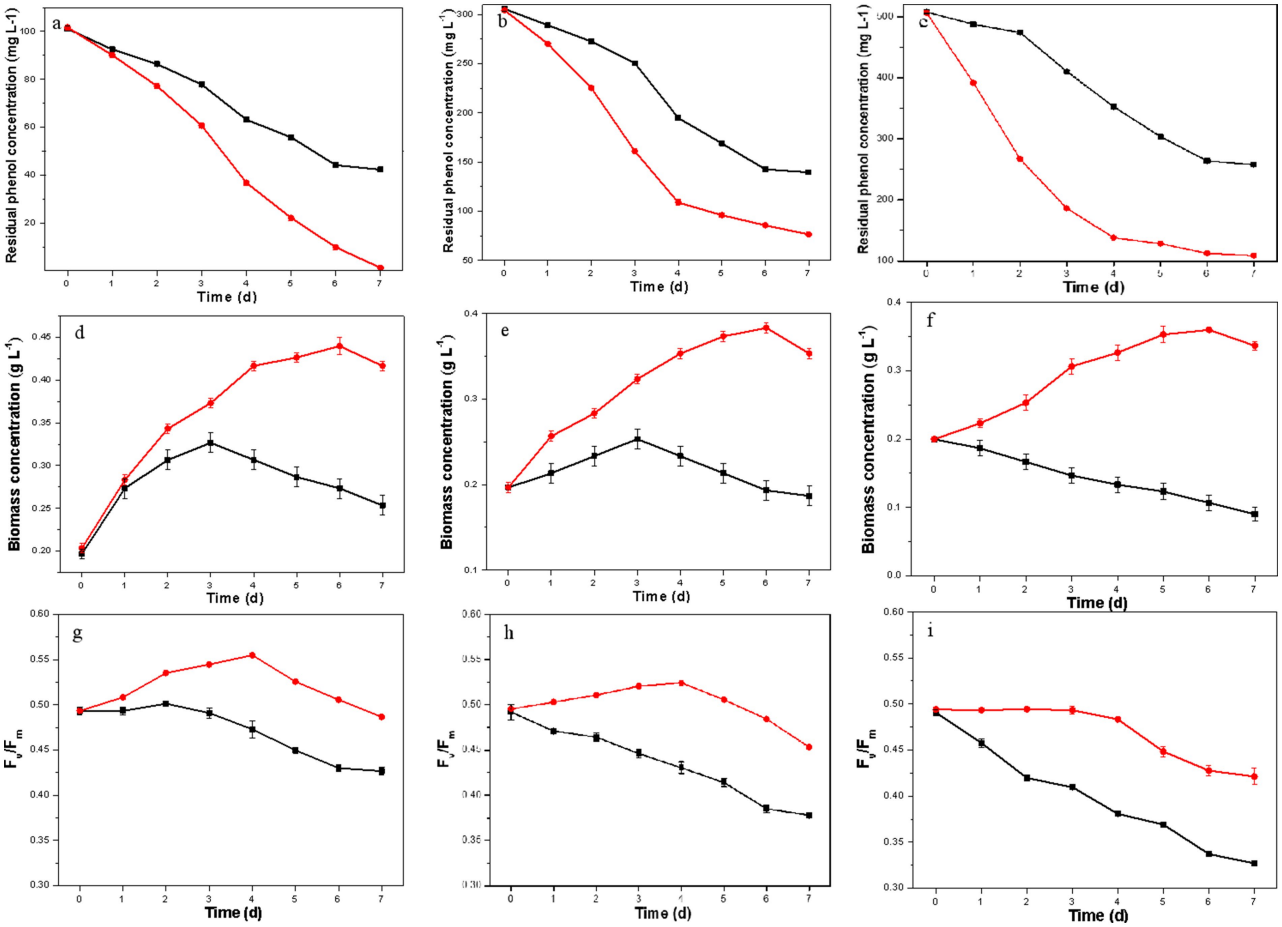
Residual phenol concentration, biomass concentration and Fv/Fm values of the original strain (black) and the resulting strain (red) under different initial phenol concentrations (**A,D,G**: 100 mg L^−1^; **B,E,H**: 300 mg L^−1^; **C,F,I**: 500 mg L^−1^).

The phenol degradation capacities of the resulting *D. salina* strain were evaluated at phenol concentrations of 100 mg L^−1^, 300 mg L^−1^, and 500 mg L^−1^. As shown in Figures 2A–C, the resulting *D. salina* completely degraded 100 mg L^−1^ phenol within 7 days, while the original strain only degraded 58.1%. At higher phenol concentrations of 300 mg L^−1^ and 500 mg L^−1^, the removal rates for the resulting strain dropped to 74.8 and 78.4%, respectively, compared to the original strain’s 54.3 and 49.2%. These findings suggest that elevated phenol concentrations create significant environmental stress, inhibiting the degradation capacity of *D. salina*. However, the phenol degradation capacity of the resulting *D. salina* strain was higher than many marine microalgal strains. Das et al. (2016) reported that the diatom BD1IITG could only degrade 39.88 and 24% of 50 and 250 mg L^−1^ phenol, respectively, after 8 d incubation. Wang et al. (2019) studied the phenol degradation ability of eight marine microalgal strains and found that *I. galbana* MACC/H59 had the best performance, which could completely degrade 100 mg L^−1^ of phenol within 4 days. These results showed that the degradation ability of phenol was enhanced, indicating that ALE improved the degradation capability to high concentration phenol.

In Figures 2D–F, the biomass concentration of the original *D. salina* initially increased before declining at phenol concentrations of 100 and 300 mg L^−1^, peaking at day 3 with maximum biomass of 0.33 g L^−1^ and 0.25 g L^−1^, respectively. At 500 mg L^−1^ phenol, biomass continuously decreased, ending at only 0.09 g L^−1^, highlighting the detrimental impact of high phenol levels on growth. In contrast, the resulting *D. salina* showed increasing biomass over time, reaching maximum concentrations of 0.44 g L^−1^, 0.38 g L^−1^, and 0.36 g L^−1^ at 100, 300, and 500 mg L^−1^ phenol, respectively. These results showed that ALE promoted the growth performance of *D. salina* under high phenol concentration, which are similar to previous reports (Li et al., 2015; Li et al., 2018; Wang et al., 2016). One evolved *Chlorella* sp. strains, AE10 was obtained after 31 cycles of ALE under 10% CO_2_. AE10 grew rapidly in 30% CO_2_ and the maximal biomass concentration of AE10 was 3.68 ± 0.08 g L^−1^, which was 2.94 times to that of the original strain (Li et al., 2015). The resulting strain was obtained after 31 cycles under 500 mg L^−1^ phenol as environmental stress. The maximal biomass concentrations of the resulting strain at day 8 were 3.40 g L^−1^ under 500 mg L^−1^ phenol, which was more than two times of that of the original strain (Wang et al., 2016).

The Fv/Fm ratio, an indicator of photosystem II efficiency and stress conditions, was also measured. As shown in Figures 2G–I, Fv/Fm values for the original *D. salina* consistently decreased across all phenol concentrations, indicating reduced photochemical efficiency due to PSII damage from phenol stress. In contrast, the resulting *D. salina* exhibited an initial increase in Fv/Fm values followed by a decrease, but overall maintained higher values than the original strain. These Fv/Fm changes were consistent with the observed phenol degradation and biomass results, further confirming that ALE enhanced the degradation ability of *D. salina* to high concentrations of phenol.

### 3.3 Antioxidative enzyme activity

High phenol concentrations induce oxidative stress and reactive oxygen species (ROS) production in microalgal cells, adversely affecting their growth (Zhou et al., 2017). To combat oxidative stress, the establishment of an antioxidative system is crucial, involving various ROS scavengers, primarily SOD, CAT and POD. These enzymes work together to mitigate ROS accumulation in microalgal cells.

In this study, antioxidant enzyme capacity was considered an important characteristic of the resulting *D. salina* strain. The antioxidative enzyme activities of the resulting *D. salina* strain and the original strain under 500 mg L^−1^ phenol were analyzed. As shown in Supplementary Figure S1A, SOD activity in both strains initially increased before declining, but the resulting strain exhibited lower values. This lower SOD activity is likely due to the original *D. salina*’s inability to quickly reduce phenol levels, requiring more SOD to convert superoxide radicals into H_2_O_2_ and O_2_. Conversely, as shown in Supplementary Figures S1B,D, the resulting strain produced more POD and less CAT than the original strain, highlighting differences in their functions. CAT primarily breaks down hydrogen peroxide to prevent oxidative damage, while POD plays a role in the antioxidant process, shielding cells from environmental stresses (Zhou et al., 2009; Liu and Pang, 2010; Mittal et al., 2012).

Oxygen free radicals generated under stress can attack polyunsaturated fatty acids in cell membranes, leading to peroxidation and the formation of harmful substances like aldehydes and ketones. Malondialdehyde (MDA) is a common indicator of membrane peroxidation and serves to assess cellular stress responses (Martins et al., 2015; Schmidt et al., 2014). As shown in Supplementary Figure S1C, the resulting *D. salina* had lower MDA levels, suggesting reduced stress from high phenol concentrations, leading to lower MDA production. While the resulting strain demonstrates enhanced tolerance, phenol can still penetrate the cell membrane during degradation, exposing it to potential membrane peroxidation effects.

Antioxidant analysis showed that resulting *D. salina* strain displayed better antioxidant potential than the original strain. If microalgal cells cannot tolerate high concentration of phenol, ROS will destroy the enzyme activities, and then inhibit microalgal growth and phenol degradation. Although the antioxidative enzyme activities were also detected, the low biomass concentration of the original *D. salina* strain indicated that it was insufficient to resist oxidative damage. The biomass concentration, phenol degradation capacity and antioxidative enzyme activity have proved that the resulting *D. salina* could tolerate high concentration phenol. However, the tolerance and degradation mechanism of that resulting *D. salina* strain against high concentration of phenol is very complex. Large duplication, rearrangement or point mutation may be happened in the evolved strain (LaPanse et al., 2021). Through the comprehensive application of a variety of omics techniques, it is possible to more systematically study the tolerance and degradation mechanism of that resulting *D. salina* strain against high concentration of phenol in the future (Kadri et al., 2023).

### 3.4 Co-culture of *Halomonas mongoliensis* and the resulting *Dunaliella salina* strain

#### 3.4.1 Phenol degradation

**Figure 3:**
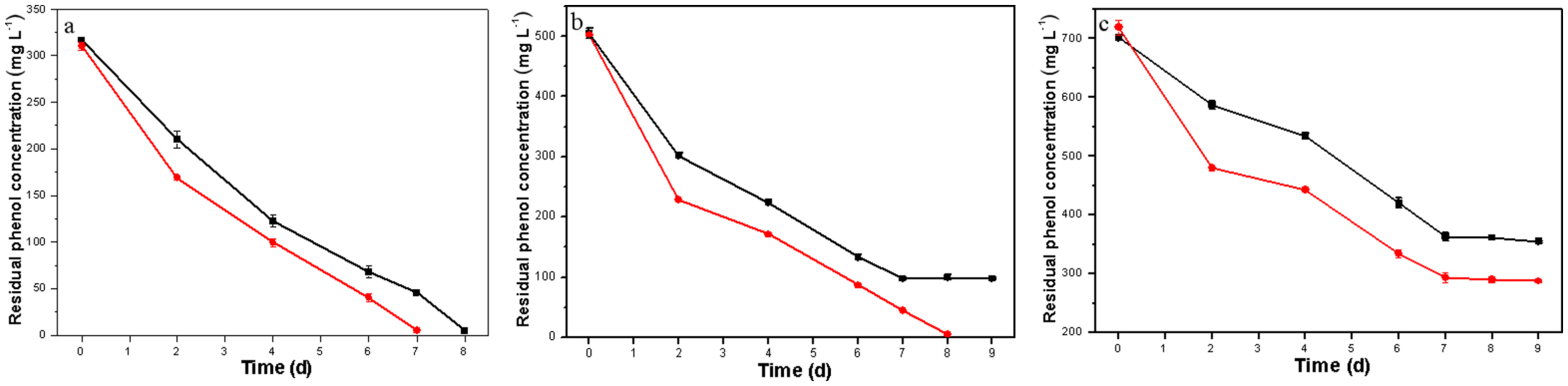
Residual phenol concentration of the co-culture of *H. mongoliensis* and the resulting *D. salina* strain (red) or the original *D. salina* strain (black) under different initial phenol concentrations (**A**, 300 mg L^−1^; **B**, 500 mg L^−1^; **C**, 700 mg L^−1^).

As illustrated in Figure 3, the co-culture of *H. mongoliensis* with either the resulting or the original *D. salina* strain exhibited superior phenol degradation capacity compared to the resulting *D. salina* strain alone (Figures 2A–C), aligning with findings from our previous study (Zhang et al., 2022). Notably, the phenol degradation capacity of the co-culture with the resulting *D. salina* strain surpassed that of the co-culture with the original strain.

At a phenol concentration of 300 mg L^−1^, the co-culture of *H. mongoliensis* and the resulting *D. salina* strain completely degraded phenol within 7 days, while the co-culture with the original *D. salina* strain achieved complete degradation in 8 days. For a phenol concentration of 500 mg L^−1^, the co-culture with the resulting *D. salina* strain managed to degrade all phenol within 8 days, whereas the co-culture with the original strain only degraded 80.6% within the same timeframe, and residual phenol remained even after extending the degradation time.

At a phenol concentration of 700 mg L^−1^, neither co-culture could completely degrade phenol within 9 days, achieving removal rates of 59.0 and 49.4%, respectively. These results indicate that high phenol concentrations impose significant environmental stress on the co-culture system, thereby diminishing its phenol degradation capacity. However, the co-culture of *H. mongoliensis* with the resulting *D. salina* strain demonstrated enhanced phenol degradation capabilities, further confirming that ALE has effectively improved the tolerance and degradation capacity of the original *D. salina* strain against high phenol concentrations.

#### 3.4.2 Fv/Fm values of *Dunaliella salina* in co-culture system

As shown in Supplementary Figure S2, the Fv/Fm values of the resulting strain in the co-culture system were consistently higher than those of the original strain. This enhancement in photosynthesis likely leads to increased O_2_ production, which is crucial for the aerobic degradation of phenol by *H. mongoliensis* (Maza-Márquez et al., 2013). The results indicate that the co-culture of the resulting *D. salina* and *H. mongoliensis* supports mutual growth, potentially facilitated by the exchange of CO_2_ and O_2_ between the two organisms (Borde et al., 2003).

However, Fv/Fm values exhibited different trends under varying initial phenol concentrations. As seen in Supplementary Figures S2A,B, Fv/Fm values initially increased before declining at phenol concentrations of 300 and 500 mg L^−1^, with final values of 0.497 and 0.461, respectively. These relatively high values suggest that *D. salina* cells maintained significant activity. In contrast, at 700 mg L^−1^ phenol, Fv/Fm values (Supplementary Figure S2C) decreased steadily throughout the culture, reaching 0.379 by the end, indicating reduced activity and diminished photochemical efficiency due to damage to PSII under heightened phenol stress. These observations align with the phenol degradation performance of the co-culture system.

#### 3.4.3 Growth of *Dunaliella salina* in co-culture system

**Figure 4:**
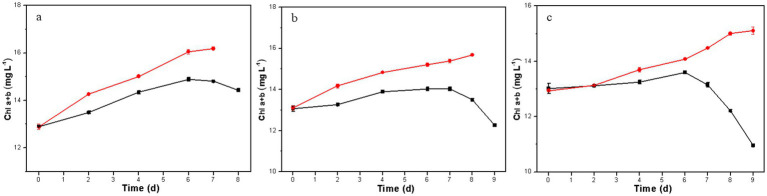
Growth of *D. salina* in the co-culture of *H. mongoliensis* and the resulting *D. salina* strain (red) or the original *D. salina* strain (black) under different initial phenol concentrations (**A**, 300 mg L^−1^; **B**, 500 mg L^−1^; **C**, 700 mg L^−1^).

The concentration of chlorophyll (a + b) in microalgal cells serves as an indicator of cell viability and biomass concentration (Russel et al., 2020). In the co-culture system, the biomass concentration of *D. salina* was indirectly assessed by measuring Chl a + b concentrations. As shown in Figure 4, the Chl a + b concentration of the resulting *D. salina* gradually increased with cultivation time under phenol concentrations of 300, 500, and 700 mg L^−1^, indicating its ability to thrive despite high phenol levels. These results were accordance with that of the resulting *D. salina*. In contrast, the original *D. salina* only showed an increase in Chl a + b at 300 mg L^−1^, while at 500 and 700 mg L^−1^, the Chl a + b content declined toward the end of the culture, indicating cell mortality under high phenol stress. Overall, high phenol concentrations are highly toxic to microalgae, significantly hindering growth and potentially leading to death (Cheng et al., 2017; Priyadharshini and Bakthavatsalam, 2017; Xiao et al., 2019). Thus, the phenol degradation in the co-culture of *H. mongoliensis* and the original *D. salina* strain was primarily due to *H. mongoliensis*, whereas the degradation in the co-culture with the resulting *D. salina* strain benefited from a synergistic effect.

## 4 Conclusion

The evolved *D. salina* strain was obtained through ALE strategy of gradually increasing phenol concentration. Compared with the original *D. salina* and *H. mongoliensis* co-culture system, the evolved *D. salina* and *H. mongoliensis* co-culture system had higher phenol degradation capability, which could effectively degrade 500 mg L^−1^ of phenol within 8 days. These results indicated that the the evolved *D. salina* and *H. mongoliensis* co-culture system had significant potential for phenol bioremediation.

## Data Availability

The raw data supporting the conclusions of this article will be made available by the authors, without undue reservation.
