# Supplementary material for: An efficient co-culture of Halomonas mongoliensis and Dunaliella salina for phenol degradation under high salt conditions
Source: Front Microbiol. 2024 Dec 11;15:1505542. doi: 10.3389/fmicb.2024.1505542 (PMC11668763; doi:10.3389/fmicb.2024.1505542)
Supplement: Supplementary file 1 [file Data_Sheet_1.docx]

**Supplementary Information**

**An efficient co-culture of *Halomonas mongoliensis* and *Dunaliella salina* for phenol degradation under high salt conditions**

Changjian Wang^a^, Haiqiao Guo^a^, Peng Yu^b,c^, Bo Huang^d,e^, Zhikun Xin^c^, Xufan Zheng^c^, Jinli Zhang^d,e,^*, Tao Tang^d,e,^*^[[1]](#footnote-0)^

^a^ChnEnergy BaoRiXiLe Energy Co., Ltd, Inner Mongolia 021000, China

^b^School of Civil and Resources Engineering, Graduate school of University of Science & Technology Beijing, Beijing 100083, China

^c^ChnEnergy New Energy Technology Research Institute Co., Ltd., Beijing 102211, China

^d^CAS Key Lab of Low-Carbon Conversion Science & Engineering, Shanghai Advanced Research Institute, Chinese Academy of Sciences, Shanghai 201210, China

^e^State Key Laboratory of Low Carbon Catalysis and Carbon Dioxide Utilization, Shanghai Advanced Research Institute, Chinese Academy of Sciences, Shanghai 201210, China

**Legends of Figure**

**Fig S1.** SOD, POD CAT activity and MDA content ofthe original strain (black) and the resulting strain (red) under the conditions of 500 mg L^-1^ phenol.

**Fig S2.** Fv/Fm values of *D. salina* in the co-culture of *H. mongoliensis* and the resulting *D. salina* strain (red) or the original *D. salina* strain (black) under different initial phenol concentrations (a, 300 mg L^-1^; b, 500 mg L^-1^; c, 700 mg L^-1^)

**Fig S1**


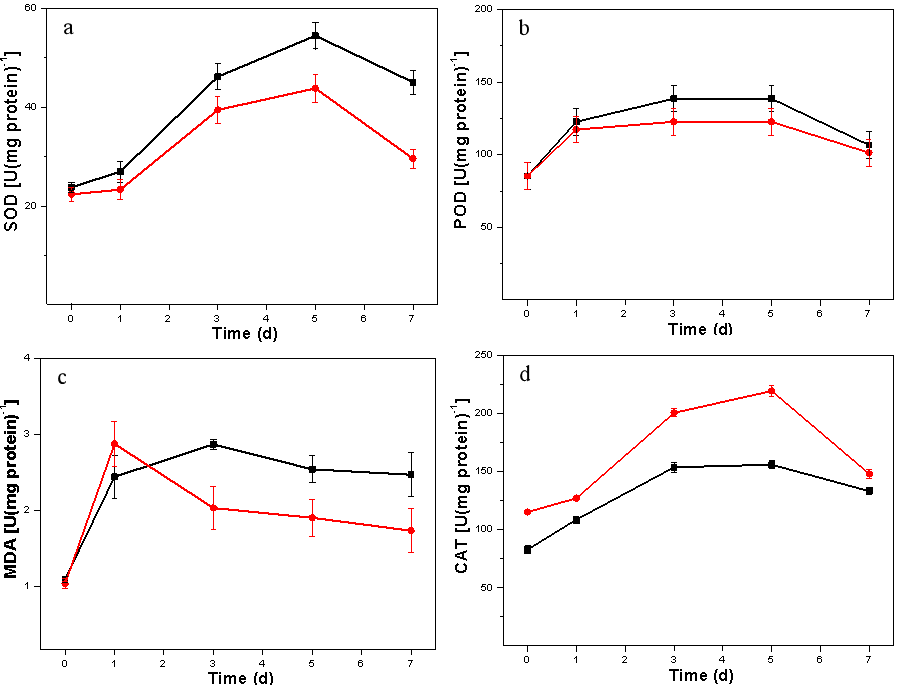


**Fig S2**


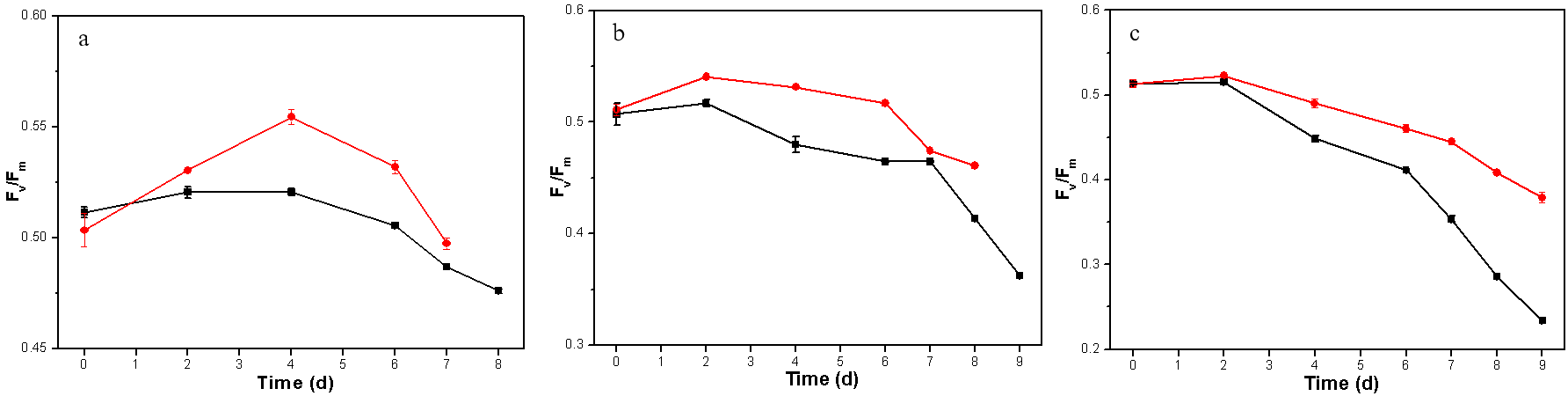


1. ^*^Corresponding author. E-mail address: [tangt@sari.ac.cn](mailto:tangt@sari.ac.cn) (Tao Tang); [zhangjinli@sari.ac.cn](mailto:zhangjinli@sari.ac.cn) (Jinli Zhang) [↑](#footnote-ref-0)
